# Spectrum of Bacterial Keratitis at a Tertiary Eye Care Centre in India

**DOI:** 10.1155/2013/181564

**Published:** 2013-08-26

**Authors:** Jayaraman Kaliamurthy, Catti Muniswamy Kalavathy, Pragya Parmar, Christadas Arul Nelson Jesudasan, Philip A. Thomas

**Affiliations:** Department of Ocular Microbiology, Institute of Ophthalmology, Joseph Eye Hospital, Trichy, Tamil Nadu 620001, India

## Abstract

*Aim*. To report the aetiological spectrum and susceptibility patterns of bacteria isolated from patients with corneal ulceration. *Method*. The microbiological data of all patients with suspected infectious corneal ulceration who presented to the ocular microbiology service at this centre between 2005 and 2012 were reviewed retrospectively. *Result*. Microorganisms were recovered from 1665 (77%) of the 2170 ulcers. Bacterial isolates accounted for 1205 of the organisms isolated. The most common bacterial pathogens isolated were various species of *Staphylococcus*, representing 777 (64.5%), followed by *Staphylococcus* spp. (148; 12.3%) and *Pseudomonas aeruginosa* (117; 9.7%). High percentages of Gram-positive bacteria were susceptible to gatifloxacin (>94%), followed by ofloxacin and moxifloxacin. Almost 90% of *Pseudomonas aeruginosa* isolates were susceptible to ciprofloxacin and moxifloxacin. Sixty-two (44%) of 140 isolates of *Streptococcus pneumoniae*, 79 (14.8%) of 534 isolates of *Staphylococcus epidermidis,* and 33 (14%) of 234 isolates of *Staphylococcus aureus* were resistant to three or more antibiotics. *Conclusion*. *Staphylococcus* spp. were the most common bacterial pathogens isolated from patients with keratitis in this setting. High percentages of Gram-positive and Gram-negative bacteria were susceptible to gatifloxacin and moxifloxacin, respectively. Interestingly, a high percentage of *Streptococcus pneumoniae* isolates were found to be resistant to three or more antibiotics.

## 1. Introduction

Bacterial keratitis is a potentially devastating corneal infection due to the possibility of rapid progression; corneal destruction may be complete in 24–48 hours with some of the more virulent bacterial aetiological agents. The spectrum of bacterial corneal pathogens is largely dictated by the local microbial flora and also by geographic and climatic factors; these probably account for disparate rates of various pathogens reported in series from different localities [1, 2]. Species of high virulence, such as *Staphylococcus aureus*, *Streptococcus pneumoniae*, *Pseudomonas aeruginosa*, and even *Neisseria meningitidis*, have been reported [3]. Coagulase-negative staphylococci (CoNS) are also being increasingly reported as the cause of bacterial keratitis in various parts of the world [1, 4–6]. The number of multiresistant CoNS identified in patients with active ocular infection has also continued to increase in recent years [4, 7]. The visual prognosis after bacterial keratitis depends on the size, locality, and depth of the ulcer, as well as on the risk factors and the bacteria isolated [8].

Bacterial keratitis is an ophthalmic emergency that needs immediate institution of treatment. In the absence of laboratory diagnosis, the initial therapy is usually broad spectrum intensive treatment. Although empirically guided therapy may suffice in cases of keratitis caused by antibiotic-susceptible bacteria, there is a risk that resistant bacteria may result in unnecessarily poor visual outcome if the microbiological diagnosis is not made [9]. Specific therapy should be based on laboratory data which identify the causative agents and provide antibacterial susceptibility results [10]. Knowledge of the microbiological pattern of bacterial keratitis will be helpful for effective management of keratitis in situations where resources are limited. The spectrum of bacteria isolated from corneal ulceration and their susceptibility patterns are described in this paper.

## 2. Materials and Methods

The study was conducted with the approval of the Institutional Ethics Committee of the authors' institution and was designed as a retrospective review of microbiological records of all patients with suspected infectious corneal ulceration who presented to the ocular microbiology service at Joseph Eye Hospital, Tiruchirappalli, Tamilnadu, southern India, between January 2005 and December 2012. 

Standard microbiological investigations [11] had been performed on material obtained under topical anaesthesia (4% lignocaine hydrochloride) by scraping the base and edges of the ulcerated part of the cornea under the magnification of a slit lamp by an ophthalmologist using a sterile Kimura platinum spatula or a sterile Bard-Parker knife. Several scrapings had been performed to obtain adequate material for direct microscopy and culture. 

For each patient, a portion of the corneal scrape material obtained had been used for direct microscopy (Gram-stained smear and lactophenol cotton blue-stained wet mount preparations), while the remaining material had also been inoculated directly onto the following media that support the growth of bacteria, fungi, and *Acanthamoeba*: sheep blood agar, Sabouraud dextrose agar and broth, and brain–heart infusion agar and broth. In addition, nonnutrient agar with *Escherichia coli* overlay had been used for clinically-suspected *Acanthamoeba* ulcers. Corneal scrapings had been inoculated onto plates of solid media by making rows of “C” streaks (two rows were made from each scraping). Brain–heart infusion agar and broth and blood agar were incubated at 37°C and were examined daily for 7 days. Sabouraud dextrose agar and the broth were incubated at room temperature (28 to 30°C) and were examined daily for 4 weeks. 

Bacterial growth obtained had been deemed significant if at least one of the following criteria had been fulfilled:

- the same bacterium was isolated on more than one solid medium;
- the bacterium isolated in culture exhibited a morphology consistent with direct microscopic findings; or
- the same organism was grown from one liquid medium and a solid medium

The bacteria isolated had been identified by standard biochemical test methods [12] and tested for their susceptibility *in vitro* to amikacin (30 *μ*g), chloramphenicol (30 *μ*g), ciprofloxacin (5 *μ*g), gatifloxacin (5 *μ*g), gentamicin (10 *μ*g), moxifloxacin (5 *μ*g), ofloxacin (5 *μ*g), and tobramycin (10 *μ*g) by the standard agar disc-diffusion method (Kirby-Bauer) using the discs obtained from Hi-Media, Mumbai, India and Mueller Hinton agar. 

## 3. Results

Two thousand one hundred and seventy patients corneal ulcers had been scraped for microbiological investigations over the period of eight years from January 2005 to December 2012. The mean age of the patients was 45.7 ± 16.6 years. Of the 2170 patients, 1274 (58.7%) were males, and 896 (41.3%) were females. Microorganisms (bacteria, fungi, and *Acanthamoeba*) were grown from 1665 (77%) of the 2170 ulcers (Table 1). Sixty-four percent of patients with culture-proven microbial keratitis had reported antecedent ocular trauma by animate and inanimate objects (Table 2). A detailed classification of the reported predisposing factors and risk factors stratified according to the microbiological agent isolated is provided in Table 2.

The mean number of corneal ulcers scraped per year from 2005 through 2012 was 271 ± 48. During this period, there were 1205 bacterial isolates from the 2170 corneal ulcers that had been scraped; 807 bacterial isolates were obtained as the sole isolate from the scraped ulcer. In 343 patients, the scraped ulcer yielded significant growth of more than one species of bacteria or growth of bacteria along with fungus (“mixed growth”) (Table 1). 

Gram-stained smears of corneal scrapings from 23 of the 807 patients (2.8%) for whom culture had yielded only a single bacterial species had not revealed presence of bacteria. Similarly, Gram-stained smears of corneal scrapings from 12 of 343 patients (3.5%) for whom culture had yielded multiple microorganisms (“mixed growth”) also had not shown presence of bacteria. In contrast, Gram-stained smears of corneal scrapings from 123 of 505 patients (24.4%) for whom there had not been any growth in culture revealed significant numbers of Gram-positive cocci or Gram-negative bacilli. 

There were 992 (82%) isolates of Gram-positive bacteria and 213 (18%) isolates of Gram-negative bacteria in culture (Table 3). The most common bacterial pathogens isolated were various species of *Staphylococcus*, accounting for 777 (64.5%) of all positive bacterial cultures, followed by* Streptococcus *spp. (148; 12.3%), *Pseudomonas aeruginosa *(117; 9.7%), *Bacillus* sp. (63; 5.2%), *Acinetobacter *sp. (56; 4.6%)), and *Aeromonas* sp. (18; 1.49%). *Enterobacter *sp., *Klebsiella pneumoniae, Serratia marcescens*, and *Flavobacterium* sp. were the least frequently isolated organisms in the series (Table 3).

Table 4 summarises susceptibility profile of the most frequent bacterial isolates recovered. Only the susceptibility test results for amikacin, chloramphenicol, ciprofloxacin, gentamicin, gatifloxacin, moxifloxacin, ofloxacin, and tobramycin were considered for analysis as they are routinely used for ocular infections in the region. More than 94% of Gram-positive bacteria were found susceptible to gatifloxacin, while a high percentage were susceptible to ofloxacin and moxifloxacin. Almost 90% of *Pseudomonas aeruginosa* isolates were susceptible to amikacin and gentamicin, while 83% were susceptible to ciprofloxacin and 82% to moxifloxacin.

When we analysed patterns of resistance to multiple antibacterials, it was found that 62 of 140 (44%) strains of *S. pneumoniae*, 79 of 534 (14.8%) *S. epidermidis* strains, 33 of 235 (14%) *S. aureus* strains, 8 of 117 (6.8) *Ps. aeruginosa *strains, 4 of 63 (6%) strains of *Bacillus* sp., and 3 of 56 (5%) strains of *Acinetobacter* sp. were resistant to three or more antibiotics. Interestingly, only one isolate of *Ps. aeruginosa* exhibited resistance to all the 8 test antibacterials (Table 5) although none of the other bacterial isolates showed resistance to all the eight antibacterials.

## 4. Discussion

Bacterial keratitis is a potentially devastating ocular infection that may occur when the corneal epithelial barrier is compromised due to injury or trauma, leading to ulceration and infiltration of inflammatory cells [13]. Infection largely involves Gram-positive *S. aureus*, *S. epidermidis*, and several *Streptococcus *and *Bacillus *spp., as well as Gram-negative bacteria such as *Ps. aeruginosa*. Immediate diagnosis and treatment are important to avoid vision-threatening outcomes, including corneal scarring or perforation.

In the present series, we found that trauma (64%) is the most common predisposing factor in our patients, a figure similar to that reported in Iran [14], Qatar [15], and Sudan [16]. Mud, dust, and soil were the most frequently reported (35.6%) followed by leaf & vegetable matter (31.6%). More or less, equal proportions of the patients in the present study reported trauma by mud and dust particles and vegetable material. Possibly during the windy season, the injury by dust particles might have been more prevalent whereas injury by vegetable matter possibly was more common during the peak season of agricultural work in this area. 

Wearing of contact lenses has been a major risk factor for bacterial keratitis in reports from Saudi Arabia, France, and Australia [3, 6, 17, 18]. In contrast to the reports cited above, contact lens wear was reported by only 12 (1%) patients in the present series. 

In the present series, microorganisms were recovered in culture from 77% of the corneal ulcers scraped. This rate of culture positivity is comparable to the 40% to 73% culture positivity rates reported in earlier studies from Australia [19], France [18], and other parts of the world [20–23].

The ability of an organism to adhere to the edge or base of an epithelial defect signatures its pathogenicity, since such an organism has the ability to invade the stroma despite adequate host defenses [24]; *S. aureus, S. pneumonia*, and *Ps. aeruginosa *are examples of bacteria with this invasive potential [25, 26]. In the present series, Gram-positive cocci accounted for 925 of 1205 (77%) bacterial isolates. This result is similar to the 69.1% [27] and 65.65% [2] reported by other Indian investigators. In other countries, Bourcier et al. [18] and Green et al. [20] reported 83% of corneal infections to be due to Gram-positive bacteria. 

The predominant Gram-positive bacterial species isolated was *S. epidermidis* (44%). In one study in Switzerland, the most commonly isolated bacterium, *S. epidermidis*, accounted for 40% of isolate [3]. Earlier studies from India [5, 27–29], Australia [6], USA, Israel, Canada, France, and New Zealand [18, 30–33] also reported *S. epidermidis* or coagulase-negative staphylococci (CoNS) to be the predominant isolate. Ly et al. [6] reported that 38% of isolates from corneal ulcers were CoNS. Butler et al. [19] reported that CoNS accounted for 23% of isolates and was the most frequent isolate recovered from corneal ulcers of elderly patients in Australia. Thus, it appears that *S. epidermidis* continues to be a very important bacterial cause of keratitis.

Only 12.3% of isolates were streptococci in our study. This is in contrast to earlier results from India [2, 34] and other developing countries such as Bangladesh and Saudi Arabia, where *S. pneumoniae *was the most common organism isolated in bacterial keratitis [35, 36]. Interestingly, in one report from Australia, only 8% of the isolates were streptococci [6]. In developing countries, the prevalence of streptococci in corneal abscesses has been linked to coexisting lacrimal drainage obstruction [6, 37]. In addition, the aetiology of the corneal ulcers may vary significantly from region to region and between rural and urban patients. In the present study and also in the studies mentioned above, the pool of patients was not analysed to differentiate rural and urban patients. 

In studies from South Florida and Hong Kong, *Ps. aeruginosa *was the most common organism isolated [38, 39], whereas *Ps. aeruginosa *accounted for only 9.7% of the isolates in our study. Our finding also differ from those of other investigators in India [29], Malaysia [40], and Beijing in China [41]. These differences may be due to regional climatic influences, number of contact lens-related keratitis cases, or the severity of cases included in each study. 

Antibiotic resistance among ocular pathogens is increasing in parallel with the increase seen among systemic pathogens and likewise may have serious consequences such as development of sight-threatening complications of keratitis, endophthalmitis, orbital cellulitis, or panophthalmitis [42–44]. In the present series, only 70% to 76% of Gram-positive organisms (*Staphylococcus* spp. and *S. pneumoniae*) were susceptible to ciprofloxacin; of these, a comparatively low percentage of *S. epidermidis* isolates were susceptible to ciprofloxacin. This is consistent with an earlier report from our centre [45]. Similarly, 69.6% of *S. epidermidis* and 73.4% of *S. aureus* isolates were susceptible to moxifloxacin, the 4th generation fluoroquinolone, whereas 86.5% of *S. pneumoniae* was susceptible to moxifloxacin. However, 94% to 98% of staphylococci and 95% *S. pneumoniae* were susceptible to gatifloxacin. These data are consistent with those reported in earlier studies [46, 47] wherein 80% of CoNS were found to be susceptible to newer-generation fluoroquinolones. Jhanji et al. [48], from India, reported a case of keratitis due to CoNS where the isolated bacterium was found to be resistant to moxifloxacin, gatifloxacin, ciprofloxacin, and cefazolin *in vitro* and also clinically resistant to moxifloxacin. It is inferred from the present study that among the fluoroquinolones tested, gatifloxacin and ofloxacin exhibited the lowest rates of resistance, and hence, gatifloxacin or ofloxacin can be recommended as first-line therapy for bacterial keratitis due to Gram-positive organisms.

Parmar et al. [45] reported that corneal ulcer healing rates with gatifloxacin were significantly higher in infections caused by Gram-positive pathogens than in those caused by Gram-negative pathogens, suggesting that gatifloxacin may be a preferred (albeit off-label) alternative to ciprofloxacin as first-line monotherapy in bacterial keratitis. The older fluoroquinolones, such as ofloxacin, ciprofloxacin, and levofloxacin, preferentially inhibit topoisomerase IV of Gram-positive bacteria, whereas the newer fluoroquinolones, such as moxifloxacin, gatifloxacin, and besifloxacin, exhibit more balanced inhibition of both DNA gyrase and topoisomerase IV [49, 50]. These newer fluoroquinolones have structural features that confer less resistance potential than their fluoroquinolone predecessors, as well as increase ocular tissue concentrations relative to organism MIC values [49, 51, 52]. Gatifloxacin and moxifloxacin are both 8-methoxy fluoroquinolones, which are less prone to resistance developing from single-step mutations, and have been shown to remain potent even in the presence of single-step resistance mutations in staphylococcal and streptococcal strains [51].

In contrast to the susceptibility results obtained for Gram-positive organisms, very high percentages of *Ps. aeruginosa* isolated were susceptible to amikacin (89.7%), gentamicin (89.7%), ciprofloxacin (82.9%), and moxifloxacin (82.3%) and lower percentages to gatifloxacin (73.5%), tobramycin (73.5%), and ofloxacin (73.5%). The percentage of ocular *Ps. aeruginosa* isolates exhibiting resistance to gatifloxacin was reported to have increased from 13.2% in a European surveillance study conducted in 2002-2003 [53]. The percentage of *P. aeruginosa* isolates showing resistance to ciprofloxacin increased from less than 1.0% of isolates obtained from 1991 to 1994 to 4.4% of those obtained from 1995 to 1998 [54, 55] and to 29% of those obtained from 2002 to 2003 [46]. Moss et al. [47] reported that 100% of their Gram-positive and Gram-negative isolates were susceptible to moxifloxacin and gatifloxacin; however, these workers recovered the isolates from the normal flora of the eyes before intravitreal injection.

In the present series, only 40% of *Ps. aeruginosa* isolate was susceptible to chloramphenicol. Ly et al. [6] reported that 100% of *Ps. aeruginosa* isolates were resistant to chloramphenicol. These results indicate that chloramphenicol should not be used routinely as the topical antibiotic of choice for corneal infection in India, a view supported by studies in Australia, Singapore, and London [6, 56, 57]. 

It must be noted that the conventional Kirby-Bauer disc diffusion method of *in vitro* antibacterial susceptibility testing may not directly apply to corneal pathogens, since the ocular antibacterial level achievable by topical administration may be considerably higher than the level attained in the ocular tissue by systemic administration, and the levels attained by topical administration may be less than the serum level of antibacterials [27]. Indeed, there have been many studies that have reported susceptible and resistant patterns of corneal pathogens based on conventional *in vitro* antibacterial susceptibility testing [4, 27, 47, 58–60], and these *in vitro* susceptible and resistant patterns have successfully guided *in vivo* treatment by these antibacterials [45, 61–64]. These results provide information that allows a clinician to make rationale choice when deciding on a primary treatment regimen which provides broad coverage for common corneal pathogens.

Table 5 shows the number and corresponding percentages of frequently isolated bacterial species that were resistant to multiple antibiotics. On the whole, 44% of *S. pneumoniae*, 15% of *S. epidermidis*, 14% of *S. aureus*, 7% of *Ps. aeruginosa*, and 6% of *Bacillus* sp. isolates were resistant to ≥3 of 8 antibacterials tested. Only 1 isolate of *Ps. aeruginosa* was resistant to all the eight antibacterials, and none of the other bacterial isolates showed resistance to all the eight antibacterials. However, certain *S. epidermidis* strains exhibited resistance to from 3 to 7 antibacterials. Recently, Moss et al. [47] reported that 20 of the 59 (34%) of the CoNS isolates cultured from 18 eyes were resistant to ≥5 of the 14 antibiotics tested. Pinna et al. [4] conducted a retrospective review (1995 to 1996) that identified 55 clinically significant strains of CoNS showing a varied spectrum of resistance patterns. One study [65] reported resistance to two or more antibiotics in 25% of the staphylococcal isolates from patients with chronic blepharitis. The current study showed that a higher proportion of *S. pneumoniae* isolates, followed by *S. epidermidis* and *S. aureus*, was resistant to multiple antibacterials. Miller et al. [66] found increased *in vitro* resistance to gatifloxacin and moxifloxacin, as well as to older fluoroquinolones, among CoNS endophthalmitis isolates recovered between 1990 and 2004. Agarwal et al. [67] reported patterns of increasing resistance of ocular bacterial isolates to moxifloxacin, gatifloxacin, and tobramycin in the subsequent years (2006 and 2007). Recently, there have also been reports of clonal spread of super bugs such as *S. pneumoniae* 19A, a highly resistant strain that has emerged in the microbiological wake left by widespread use of pneumococcal vaccines [68]. Resistance to multiple antibiotics might possibly represent a response to prolonged treatment [4]. This is of concern because the spread of such strains in hospitals could be alarming in immunocompromised patients. It is recommended to perform antibiotic susceptibility testing in all cases of clinically significant ocular infections caused by these organisms.

Microbial resistance to antibiotic agents is becoming increasingly prevalent in ocular infections [69]. The past 2 decades have witnessed changes in antibiotic susceptibility patterns on a worldwide basis. Guidelines that have been developed to help slow the escalation of systemic antibiotic resistance and encourage prudent use of antibiotic agents also apply to the management of ocular infections. Clinicians should prescribe antibiotic agents only when clearly indicated and should order susceptibility testing whenever possible to prescribe the most appropriate agent [69]. The excessive use of antibiotic agents is a primary cause of resistance. In addition, physicians should select agents that have rapid bactericidal activity, high attainable concentrations at the site of infection compared with the organism MIC, a relatively low incidence of antibacterial resistance, and a broad spectrum of activity. 

## Figures and Tables

**Table 1:** Aetiological spectrum of microbial keratitis over an 8-year period (2005–2012) at a tertiary eye care facility in India.

Year	Bacterial only	Fungal only	Mixed (bacterial + fungal)	Others*	No growth	Total
2005	95	89	19	5	120	328
2006	86	80	23	0	119	308
2007	109	105	38	2	72	326
2008	108	44	36	6	63	257
2009	115	64	44	1	56	280
2010	88	51	69	0	27	235
2011	121	35	56	4	26	242
2012	85	25	58	4	22	194

Total	807^+^	493	343^++^	22^+++^	505	2170

*Acanthamoeba sp. & Nocardia sp.

^+^In some patients, more than one bacteria was isolated.

^++^In some patients, more than one bacteria was isolated in addition to fungi.

^+++^Nocardia accounted for 13 isolates.

**Table 2:** Putative risk factors for culture positive microbial keratitis (total number of culture proven microbial keratitis = 1665).

	Total	Results of culture				
		Bacterial only	Fungal only	Mixed (bacterial + fungal)	Others*	No growth
Number of patients	2170	807	493	343	22	505
Trauma	**1065 (64)**					
Mud, dust, and soil	379 (35.6)	125	114	72	2	66
Leaf & vegetable matter	337 (31.6)	75	121	53	3	85
Stick	134 (12.6)	47	36	12	1	38
Stone	59 (5.5)	22	12	8	1	16
Insect	50 (4.7)	25	14	6	0	5
Finger nail	43 (4.0)	16	12	4	1	10
Wood piece	20 (1.9)	5	6	3	1	5
Metal (iron)	19 (1.8)	6	2	3	0	8
Animal tail, horn	9 (0.8)	3	3	2	1	0
Glass piece	9 (0.8)	1	1	1	0	6
Ball	6 (0.6)	1	1	1	0	3
Contact lens wear	**16 (1.0)**	6	0	2	0	8
Use of eye drops (antibiotics)	**322 (19.3)**	84	99	33	2	104
Use of traditional eye medicine (oil, leaf juice, milk, etc.)	**282 (16.9)**	64	135	81	0	2

**Table 3:** Bacterial isolates recovered from patients with corneal ulceration over an 8-year period (2005–2012) at a tertiary eye care facility in India.

Bacterium	2005 *n* (%)	2006 *n* (%)	2007 *n* (%)	2008 *n* (%)	2009 *n* (%)	2010 *n* (%)	2011 *n* (%)	2012 *n* (%)	Total *n* (%)
*Staphylococcus epidermidis *	48 (37.5)*	38 (31.7)*	73 (43.7)*	81 (54)*	75 (46.8)*	87 (51.8)*	85 (45.4)*	47 (37.6)*	534 (44)
*Staphylococcus aureus *	03	10	26	21	28	35	61	51	235 (19.5)
*Staphylococcus saprophyticus *	00	04	00	01	02	0	1	0	8 (0.6)
**Staphylococci total**	**51**	**52**	**99**	**103**	**105**	**122**	**147**	**98**	**777 (64.5)**
*Streptococcus pneumoniae *	27	24	27	13	14	14	12	9	140 (11.6)
Viridans streptococci	0	0	0	0	08	0	0	0	8 (0.6)
**Streptococci total**	**27**	**24**	**27**	**13**	**22**	**14**	**12**	**9**	**148 (12.3)**
**Total Gram-positive cocci total**	**78**	**76**	**126**	**116**	**127**	**136**	**159**	**107**	**925**
*Bacillus *species	21	13	07	05	04	3	2	8	63 (5.2)
*Corynebacterium diphtheriae*.	00	00	03	00	00	1	0	0	4 (0.3)
**Gram-positive bacilli total**	**21**	**13**	**10**	**5**	**4**	**4**	**2**	**8**	**67**
**Gram positive organisms total**	99	89	136	121	131	140	161	115	992
*Pseudomonas aeruginosa *	15	23	20	17	10	18	11	3	117 (9.7)
*Acinetobacter *species	10	08	04	05	11	5	9	4	56 (4.6)
*Aeromonas *species	02	00	04	01	04	2	4	1	18 (1.49)
*Enterobacter *species	0	0	02	04	02	0	0	0	08 (0.6)
*Klebsiella pneumoniae *	01	00	01	02	02	3	2	1	12 (0.99)
*Serratia *species	01	00	00	00	00	0	0	0	01 (0.08)
*Flavobacterium *species	0	0	0	0	0	0	0	1	01 (0.08)
**Gram negative total**	**29**	**31**	**31**	**29**	**29**	**28**	**26**	**10**	**213**

Total isolates	128	120	167	150	160	168	187	125	1205

*n*: number of isolates; %: corresponding per cent; *% in respective year.

**Table 4:** Frequent bacterial isolates and percentage of strains susceptible to antibacterial agents in bacterial keratitis at a tertiary eye care facility in India (2005–2012).

Organism (number of isolates)	Antibacterial agents							
	AK *n* (%)	C *n* (%)	CF *n* (%)	G *n* (%)	GF *n* (%)	MO* *n* (%)	OF *n* (%)	TB *n* (%)
*Staphylococcus epidermidis* (534)	477 (89.3)	429 (80.3)	376 (70.4)	436 (81.6)	501 (93.8)	312^1^ (69.6)	430 (80.5)	426 (79.8)
*Staphylococcus aureus* (235)	224 (95.3)	199 (84.7)	180 (76.6)	199 (84.7)	230 (97.9)	163^2^ (73.4)	195 (83.0)	203 (86.4)
*Streptococcus pneumoniae* (140)	53 (37.8)	115 (82.1)	106 (75.7)	43 (30.7)	133 (95)	77^3^ (86.5)	121 (86.4)	60 (42.8)
*Bacillus *spp. (63)	59 (93.6)	57 (90.5)	47 (74.6)	58 (92.0)	61 (96.8)	22^4^ (75.9)	62 (98.4)	59 (93.6)
*Pseudomonas aeruginosa* (117)	105 (89.7)	47 (40.2)	97 (82.9)	105 (89.7)	86 (73.5)	65^5^ (82.3)	86 (73.5)	86 (73.5)
*Acinetobacter *sp. (56)	52 (92.8)	46 (82.14)	48 (85.7)	50 (89.28)	52 (92.8)	35^6^ (92.1)	43 (76.8)	43 (76.8)

*Due to unavailability of moxifloxacin discs in the year 2005, the total numbers of isolates tested against moxifloxacin are different from the numbers of isolates tested against other antibiotics. Hence, the total number of isolates tested for moxifloxacin = ^1^448; ^2^222; ^3^89; ^4^29; ^5^79; and ^6^38.

*n*: number of isolates susceptible to the antibiotic. %: corresponding per cent.

AK: amikacin; C: chloramphenicol; CF: ciprofloxacin; G: gentamicin; GF: gatifloxacin; OF: ofloxacin; MO: moxifloxacin; and TB: tobramycin.

**Table 5:** Resistance patterns of frequent bacterial isolates recovered from corneal ulceration over an eight-year period (2005–2012) at a tertiary eye care facility in India.

Resistance to	Organism					
	*S. epidermidis* *n* (%)	*S. aureus* *n* (%)	*S. pneumoniae* *n* (%)	*Bacillus* spp. *n* (%)	*Ps. aeruginosa* *n* (%)	*Acinetobacter* sp. *n* (%)
8 antibiotics	0	0	0	0	1 (0.8)	0
7 antibiotics	1 (0.2)	1 (0.4)	0	0	0	0
6 antibiotics	4 (0.7)	1 (0.4)	1 (0.7)	0	2 (1.7)	0
5 antibiotics	13 (2.4)	2 (0.8)	1 (0.7)	0	0	1 (0.8)
4 antibiotics	22 (4.1)	6 (2.5)	15 (10.7)	1 (1.6)	3 (2.6)	0
3 antibiotics	39 (7.3)	23 (9.8)	45 (32.1)	3 (4.8)	2 (1.7)	2 (1.7)
2 antibiotics	83 (15.5)	29 (12.3)	39 (27.8)	6 (9.5)	19 (16.2)	3 (5.3)
1 antibiotic	128 (23.9)	48 (20.4)	21 (15)	7 (11.1)	63 (53.8)	11 (19.6)
0 antibiotic	244 (45.7)	125 (53.2)	18 (12.8)	46 (73)	27 (23)	39 (69.6)

Total isolates tested	534	235	140	63	117	56

*n*: number of isolates that showed resistance, %: corresponding per cent.

Selected antibiotics: amikacin, chloramphenicol, ciprofloxacin, gentamicin, gatifloxacin, moxifloxacin, ofloxacin, tobramycin.
